# Reflections, resilience and recovery: a qualitative study of Covid-19’s impact on an international adult population’s mental health and priorities for support

**DOI:** 10.14324/111.444/ucloe.000041

**Published:** 2022-12-01

**Authors:** Keri Ka-Yee Wong, Kimberly Loke, Kyleigh Marie Kai-Li Melville

**Affiliations:** 1 Department of Psychology and Human Development, University College London, London, UK; 2 Faculty of Education, University of Cambridge, Cambridge, UK

**Keywords:** Covid-19, mental health, behavioural change, qualitative, financial burden, support

## Abstract

The impact of the coronavirus 2019 (Covid-19) pandemic on different countries and populations is well documented in quantitative studies, with some studies showing stable mental health symptoms and others showing fluctuating symptoms. However, the reasons behind why some symptoms are stable and others change are under-explored, which in turn makes identifying the types of support needed by participants themselves challenging. To address these gaps, this study thematically analysed 925 qualitative responses from five open-ended responses collected in the UCL-Penn Global COVID Study between 17 April and 31 July 2021 (Wave 3). Three key themes that comprised 13 codes were reported by participants across countries and ages regarding the impact of Covid-19 on their health, both mental and physical, and livelihoods. These include: (1) *Outlook on self/life*, (2) *Self-improvement*, and (3) *Loved ones (friends and family)*. In terms of support, while 2.91% did not require additional support, 91% wanted support beyond financial support. Other unexpected new themes were also discussed regarding vulnerable populations suffering disproportionately. The pandemic has brought into sharp focus various changes in people’s mental health, physical health and relationships. Greater policy considerations should be given to supporting citizens’ continued access to mental health when considering pandemic recovery.

## Introduction

We are not all in the same boat.
We are all in the same storm.
Some are on super-yachts.
Some have just the one oar.
     - Damian Barr (2020)

The novel coronavirus 2019 (Covid-19) pandemic took the world by surprise in early 2020 [1], forcing many of us to reassess our priorities and rethink the future. It did not take long for countries and individuals to learn that we are in the same storm but indeed, not in the same boat. The Covid-19 pandemic has brought into sharp focus society’s disparities at all levels. Health has become a key topic of everyday conversations as we grapple with the precarity of ‘good health’ – both physical and mental health. Pandemic policies, restrictions and repeated lockdowns – although varying in length and severity by country – have undoubtedly impacted people’s livelihoods and outlook on life, some for the short-term, others for much longer [2–6]. Rippling effects are still being observed at the global economic level and in key sectors such as healthcare and education arguably for years to come [7]. The last two years have seen an ever-widening gap between the developing and developed world in access to vaccines, contrasts between governments’ action and inaction and the rising global death toll. To overcome this pandemic – and future pandemics to come – the international community must come together in solidarity to fight this virus.

One way of coming to a shared resolution is to understand the impact of the pandemic on people’s lives and the support they might need. At the time of writing this article, international media coverage has primarily focused on the economic and financial costs brought on by the Covid-19 pandemic. Whether this is a key focus on people’s minds is less clear. At the time of writing this article, additional European lockdowns over the winter of 2021 are being put in place (e.g., Austria, Germany, the Netherlands) and the UK has reinstated mandatory face masks in shops and public transport. The costs of partial and full lockdowns on businesses as well as the rapid circulation of the Omicron variant has also meant that countries such as New Zealand, which has maintained a ‘zero covid policy’, have had to also accept that Covid-19 is here to stay. Parts of Asia which rely heavily on tourism have been rebuked for their long and stringent quarantine rules (21 days to 1 month), yet have maintained their stance in slowly imported cases. And as the world rolls-out potential Covid-19 antiviral pills, clinical trials on needle-less vaccines, and booster jabs for the population – still, less than half of the world is currently vaccinated (42.4%), with only 6% of the African population having received the first dose. As policymakers worldwide continue to react to, rather than staying on top of new variants, the pandemic by prioritising the financial and economic gains over more punitive public health safety measures, scientific evidence and data are becoming increasingly vital in informing current and future public health policy and recovery strategies.

In particular, research on the impacts of Covid-19 on mental health since the start of the pandemic has seen exponential growth. Numerous quantitative studies from different countries have reported on the impacts of the Covid-19 pandemic on the general population’s mental health [8–10] but many more studies have focused on specific populations including: healthcare professionals and providers [11,12], educational professionals [13], patients with existing mental health conditions [14], young children and adolescents [15–17] and young adults and undergraduates [18,19] to name a few. While most studies are cross-sectional or focused on the first 12–18 months of the pandemic [20], a handful of studies have also continued beyond that to report on the longer-term health impacts of the Covid-19 pandemic on health [21]. Studies on the stability and changes in rates of mental health symptoms while informative do not by design offer insight into the underlying *reasons* for the stability and change as well as *potential solutions* in the way that is captured by qualitative studies. As such, qualitative studies are immensely valuable in generating a more in-depth understanding of how populations are faring during the pandemic where the day-to-day environment in which they live has changed greatly.

To date, qualitative studies examining the impact of the Covid-19 pandemic on sub-populations’ mental health have uncovered a variety of experiences. In one semi-structured telephone and video interview study of older adults in the UK aged 70 years and above (n = 20) conducted between May and September 2020, researchers found that ‘fears for mortality’, ‘grieving normal life’ and ‘concerns for the future’ were identified as potential threats to this group’s mental well-being [22]. Participants spoke about coping activities and behaviours including ‘adopting a slower pace of life’, ‘maintaining routine’, ‘socialising’ and ‘using past coping skills’ as protective factors of mental health. Unsurprisingly, participants also drew on personal experience to manage the fear and uncertainty brought on by the pandemic and used the lockdown to reflect or organise end-of-life affairs. These themes were consistent with another study of a geriatric population (60+ years) in Buenos Aires conducted during a similar period (April–July 2020), where distress, anxiety, anger, uncertainty, exhaustion and expressed fear of contagion from themselves and their loved ones were also key themes [23]. In addition, this study identified individuals who lived alone, lived in small and closed environments, with weak relational networks, or limited access to technologies were more vulnerable.

In other qualitative studies of young children and families, Sullivan et al. [24] interviewed Irish families (n = 48) and found clear negative impacts of Covid-19 restrictions on young people’s mental well-being. These included negative feelings of social isolation, depression, anxiety and increased maladaptive behavioural changes, such as clinginess in younger children, were common. Families with children with autism spectrum disorders (ASD) in particular, reported increased mental health difficulties. These findings are consistent with quantitative studies of UK families with special education needs children and disabilities in the UK [25] and families even with typically developing children [17]. Drawing on these studies, it is evident that people who live alone and those with different family structures should be taken into consideration when developing appropriate support.

Studies of individuals living with pre-existing mental health conditions paint a similar picture. Taking a co-production participatory approach, Gillard et al. [26] conducted an online video interview study between 18 May and 8 July 2020 and found that mental health difficulties were further exacerbated in those with pre-existing mental health conditions. Specifically, some people struggled with staying connected and accessing mental health support and services, while others found new ways to cope and stay connected with the community. For some people, access to mental health care through technology was possible, but for others, there were substantial barriers. Specifically, individuals from black and minority ethnic groups reported heightened pandemic-related anxiety, stigma and racism that further impacted their mental health. These contrasting experiences highlight the need for a better understanding of providing targeted and effective support for sub-groups in the population.

Global studies of healthcare professionals and medical staff are also fairly consistent. In a semi-structured interview study of Iranian healthcare professionals (n = 97) conducted between 10 March and 4 July 2020, four themes were highlighted by this group: ‘Working in the pandemic era’, ‘Changes in personal life and enhanced negative affect’, ‘Gaining experience, normalisation and adaptation to the pandemic’ and ‘Mental health considerations’ [27]. Similar themes were reported by Swedish frontline doctors (n = 20) working in intensive care units (ICU) during Spring 2020: ‘Professionalism in work-life’ (adaption, the patient’s welfare, insecurity and security), ‘Community spirit’ (responsibility and contribution), and ‘Institutional organisation’ (the role of management, loss of freedom and information) [28]. This is not dissimilar to the reports of Italian healthcare professionals (n = 19), where individual motivations/ethics, interpersonal relationships and support, and work/organisational leadership and messaging were identified as risk and protective factors during the pandemic [29]. Although individuals from the same occupation group were being interviewed, the resultant themes from different countries were more similar than different, suggesting that the impacts of the global pandemic may be more universal for some groups than country specific. However, as interview questions may differ across studies and with a focus on just one small group of individuals absent of comparison groups, these data are limited in that comparisons on qualitative experiences across different occupational groups or countries are not possible.

The current qualitative study aims to understand the positive and negative impacts of the Covid-19 pandemic on people’s experiences, perspectives and livelihoods. A key question is to identify whether there are country-specific and/or universal themes that people have raised and how they may inform international policies in pandemic recovery plans. To the best of our knowledge, few existing studies have looked at the varying socioeconomic and emotional impacts of Covid-19 across multiple countries, and even fewer studies have aimed to understand country similarities and contrasts in people’s perceptions and need for support post-pandemic. Should individuals voice the same needs regardless of whether they are in the same country, this would suggest that universal strategies are needed, while country-specific needs may better serve country-specific recovery plans. As such, our study tests three main hypotheses and one open-ended hypothesis:

1. How has people’s health (mental and physical) and livelihoods been negatively impacted by the Covid-19 pandemic? We hypothesise that the impacts of the pandemic have primarily been negative (e.g., Covid-19-related anxiety, staying connected, mental health access) with some positive impacts as well.
2. How do the above effects differ by country, gender, age and socioeconomic status?
3. What support do people need? We hypothesise that there will be country-specific and universal needs, and different needs for different groups of participants.
4. We also predict there will be differences in experiences and solutions, hence unexpected themes may also be generated and shed light on future research directions where people’s environmental conditions have changes.

## Methods

### Participants

Over 2300 adult volunteers took part in a 30-minute online survey in Wave 1 (April–July 2020), 1806 in Wave 2 (October 2020–January 2021) and 925 in Wave 3 (April–July 2021). Participants were recruited via online advertising of the study, university lists, charity lists, LinkedIn, Twitter, Instagram and word-of-mouth. All adults aged 18 years and above with access to the study website GlobalCOVIDStudy.com could take part. The survey was available in English and seven other languages (Greek, Italian, Spanish, Chinese Traditional, Chinese Simplified, French, German). Forward translations were first conducted by Google translate and cross-checked and corrected by one or more native speakers. This study was pre-registered (https://osf.io/4nj3g/ on 17 April 2020) and ethical approval was obtained from the IOE (Institute of Education), UCL’s Faculty of Education and Society (University College London, UK) Ethics and Review Committee on 8 April 2020 (REC 1331; [1]). Informed consent was sought from participants at the start of the 30-minute online Qualtrics survey and at subsequent follow-ups, with opt-out options available throughout. Participants could skip the question if they did not wish to answer it.

The analytic sample for this study is from Wave 3 only and consists of qualitative responses from 925 participants (females = 75.7%, M = 0.81, standard deviation [SD] = 0.51 years) from the United Kingdom (47.8%), the United States (11.6%), Italy (6.3%), Greece (5.5%), Hong Kong (3.0%), Canada (2.6%) and China (2.1%) (see Appendix 1). Additional participant information can be found in Table 1.

**Table 1.** Participant characteristics

Characteristic	n	%
Participant gender		
Male	207	22.4%
Female	701	75.7%
Other	16	1.7%
Missing	2	0.2%
Current employment status		
Undergraduate student (full/part-time)	61	6.6%
Postgraduate student (e.g., MSc/MA) (full/part-time)	39	4.2%
Graduate student (e.g., PhD/DPhil) (full/part-time)	194	21.0%
Working (paid employee)	380	41.0%
Working (self-employed)	77	8.3%
Not working	50	5.4%
Retired	49	5.3%
Prefer not to answer	2	0.2%
Unemployed	22	2.4%
Furloughed	10	1.1%
In between jobs	11	1.2%
Missing	31	3.3%
Estimate of entire household income (pre-tax) in the previous year		
Less than £10,000	90	9.7%
£10,000 to £19,999	108	11.7%
£20,000 to £29,999	91	9.8%
£30,000 to £39,999	84	9.1%
£40,000 to £49,999	68	7.3%
£50,000 to £59,999	76	8.2%
£60,000 to £69,999	57	6.2%
£70,000 to £79,999	40	4.3%
£80,000 to £89,999	34	3.7%
£90,000 to £99,999	41	4.4%
£100,000 to £149,999	76	8.2%
£150,000 or more	96	10.4%
Missing	55	7.1%

### Design

The current qualitative study is based on five open-ended questions embedded in a larger battery of questionnaires administered as part of the UCL-Penn Global COVID Study [1]. This study was conducted and reported in line with the Consolidated Criteria for Reporting Qualitative Research (COREQ) where appropriate. All questions gauged the impact of the Covid-19 pandemic on the general population’s mental health, livelihoods, and need for future support as of 17 April to 31 July 2021.

### Measures

The five open-ended qualitative questions asked to better understand the impact of the Covid-19 pandemic on people’s lifestyle, behaviours and mindset and, importantly, potential support that individuals and families would need in the next 6 months were:

1. Reflecting on the past year, how has Covid-19 changed your lifestyle, behaviours and thinking for the BETTER? (Q52)
2. Reflecting on the past year, how has Covid-19 changed your lifestyle, behaviours and thinking for the WORSE? (Q73)
3. Did you learn anything new about yourself or others during the pandemic? (Q74)
4. Reflecting on the past year, name a few things you did to better cope and become more resilient during the pandemic. (Q71)
5. Thinking ahead, what support would you/your family need in the next 6 months to thrive and recover from the pandemic? (Q72)

### Data analysis

Braun and Clark’s [30] six-step thematic analysis were conducted on our qualitative data. Three researchers (KW, KM, KL) independently conducted the steps to minimise bias as best as possible and met as a team when discussing discrepancies in coding. The following steps were conducted in an iterative manner:

1. Familiarising ourselves with the data (all researchers)
2. Generating initial codes systematically (consensus on coding scheme)
3. Reviewing codes and cross-checking for inter-rater reliability between codes
4. Adding new codes and refining codes
5. Searching for themes
6. Defining and naming themes

Data were analysed using IBM SPSS Statistics for Windows, version 21.0 (IBM Corp., Armonk, NY, USA) and Microsoft Excel. Data were stripped of basic participant background information (e.g., sex, age, country of origin, socioeconomic status) to minimise researcher bias. Missing data for each question were coded as −99 (no answer) or −999 (scored neutrally as difficult to interpret) and described in Appendix 2.

### Establishing inter-rater reliability

To ensure that all coders were consistent in applying the same codes across all responses, data were first reviewed independently by each researcher to identify example quotes and respective codes and repeated through subsequent iterative meetings. Inter-rater reliability (IRR) checks were conducted between researchers KL and KM with KW providing a third-party opinion, first on responses from Q52 to develop a set of 13 refined codes (see Appendix 3 for detailed IRR process and notes).

Briefly, 13 initial codes were established after all three coders independently reviewed the data: mental health, outlook on life, loved ones, sedentary behaviour, self-improvement, loss of motivation, optimism about the future, financial security, Covid-19 policy, access to services, loss, virtual living, frustration towards others, distrust in media/government and does not need support (see Table 2). Next, KL and KM coded Q52 independently against the 13 initial codes and took notes after each round of independent coding to document potential issues for group discussion. Aiming for an IRR above 80%, a random number generator identifying 10% of coded responses in Q52 resulted in a low IRR threshold in the first meeting (64%) and second meeting (75%), but a high reliability by the third meeting, (81.2%). At each iteration, discordant codes were discussed between researchers and addressed in subsequent iterations. After the third meeting, the team coded the rest of the responses in the dataset. Non-English responses (e.g., Italian, French, Greek) were translated through Google Translate, taking care that translations of smaller chunks of inputted text resulted in more accurate translations.

**Table 2.** Thirteen codes derived from participant responses

Code	Code name
1	Mental health (perceptions, feelings, and cognitions)
2	Outlook on self/life
3	Loved ones (friends, family)
4-	Sedentary behaviours (inactive, decrease in behaviours)
4+	Self-improvement (active, increase in behaviours)
5-	Loss of motivation/pessimism about the future
5+	Motivation/optimism about the future
6	Finances/work/studies
7	Covid-19 policies
8	Access to services/support
9	Loss/bereavement
10	Virtual living/virtual events
11	Frustration towards others
12	Distrust in media and government
13	Does not need support
−999	Scored neutrally as difficult to interpret
−99	Missing

## Results

**Figure 1:**
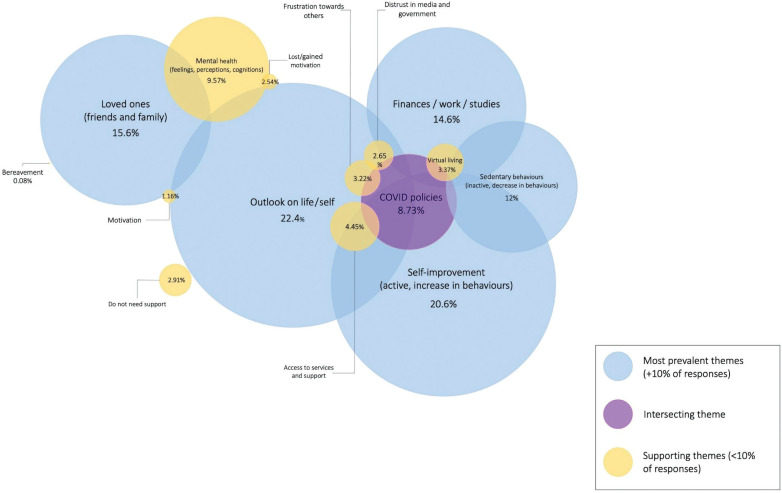
Visual summary showing the relationship between the 13 codes and the extent of overlapping themes across the data set. The size of the circles is relative to their prevalence rates in the dataset, whereby a larger circle represents higher prevalence (e.g., the relative size of the circles was made by setting the length and width of the circles equivalent to their prevalence rate).

Thematic analysis across our dataset revealed three key themes comprising 13 codes of varying degrees of overlap: (1) Outlook on self/life, (2) Self-improvement, and (3) Loved ones (friends and family). See example quotes in Table 3 and a visual representation of overlap themes across questions in Figure 1.

**Table 3.** Example and prevalence of codes across five questions from 925 participants (total 4625 responses)

Code	Frequency (n = 4625)	Proportion	Examples
1: Mental health (perceptions, feelings, and cognitions)	443	9.57%	‘My mental health was fine prior to the pandemic, but now it’s certainly not’ ‘Feeling lonely and not talking to friends when I feel sad’ ‘Slightly more wary of acquaintances less open to new relationships more guarded’
2: Outlook on self/life	1036	**22.4%**	‘Live life and enjoy yourself’ ‘slower pace of life’ ‘live more present’ ‘worry less’ ‘I’ve learned to accept myself as I am, instead of chasing some ideal version of myself’
3: Loved ones (friends, family)	726	**15.6%**	‘I feel closer to my husband and have really enjoyed seeing him more’ ‘Appreciating others around me’ ‘Feel closer to family’
4-: Sedentary behaviours (inactive, decrease in behaviours)	555	12%	‘I feel worried that I may have become more sedentary and make less effort to go out’ ‘Antisocial’ ‘Hermit’
4+: Self-improvement (active, increase in behaviours)	957	**20.6%**	‘I’ve been trying to get better about spacing things out and doing more than usual when I know I’m feeling good since bad days will come’ ‘Started doing weight-lifting, learning ukulele, not judging my food cravings’
5-: Loss of motivation/pessimism about the future	65	1.38%	‘Losing interest in things’ ‘Hard to stay active’
5+: Motivation/optimism about the future	54	1.16%	‘Being hopeful about the future, trying to find jobs and opportunities that interest me to pursue after my studies’
6: Finances/work/studies	679	14.6%	‘Lowered job prospects’ ‘Workload’ ‘Partner needs to find a job’
7: Covid-19 policies	404	8.73%	‘No more lockdowns … lifting of international travel restrictions’
8: Access to services/support	206	4.45%	‘I want other people to get vaccinated’ ‘It has improved my access to health services because I can access services from home instead of having to find adequate public transportation’
9: Loss/bereavement	4	0.08%	‘I lost my nan … and I feel disappointed about all the things we can’t do’
10: Virtual living/virtual events	156	3.37%	‘videoconferencing and dialoguing with colleagues’
11: Frustration towards others	149	3.22%	‘More people than I thought turned out to be stupid … Anti vaccine and such’
12: Distrust in media and government	123	2.65%	‘Stopped watching government covid broadcasts, and the news’
13: Does not need support	135	2.91%	‘Nothing additional to what we already have’
−999: Scored neutrally as difficult to interpret	363	7.84%	‘no’ ‘yes’ ‘it has/has not’
−99: Missing	1040	22.4%	Blank

Note: Values in **bold** show the top three most prevalent themes.

To investigate which codes had the greatest proportional overlap with one another, descriptive tables were generated for all five questions and yielded a total of 488 unique combinations of codes (e.g., codes 1, 2, 3). The following table shows the distribution of codes for each question (see Appendix 4 for example codes) and the percentage of overlap between codes across the five questions (Table 4).

**Table 4.** Distribution of codes for each question (all n = 925)

Code	Q52	Q71	Q72	Q73	Q74	Percentage of overlap (n = 488)
1: Mental health (perceptions, feelings, and cognitions)	7.14%	3.03%	5.73%	6.49%	3.24%	21.1%
2: Outlook on self/life	15.0%	17.3%	6.60%	27.0%	46.1%	36.6%
3: Loved ones (friends, family)	13.7%	15.4%	10.8%	18.2%	12.5%	41.3%
4-: Sedentary behaviours (inactive, decrease in behaviours)	9.19%	5.84%	0.75%	42.0%	1.83%	33.1%
4+: Self-improvement (active, increase in behaviours)	24.0%	53.3%	13.6%	8.76%	4.00%	32.7%
5-: Loss of motivation/pessimism about the future	0.75%	0%	0%	6.06%	0.21%	7.58%
5+: Motivation/optimism about the future	2.59%	0.10%	0.97%	0.32%	1.73%	7.58%
6: Finances/work/studies	24.6%	7.46%	20.6%	14.1%	6.60%	34.0%
7: Covid-19 policies	10.0%	2.70%	17.2%	10.2%	3.57%	28.4%
8: Access to services/support	3.46%	4.11%	12.2%	2.05%	0.43%	15.7%
9: Loss/bereavement	0%	0%	0.21%	0.10%	0.10%	1.02%
10: Virtual living/virtual events	6.06%	6.70%	0.43%	1.40%	2.27%	13.9%
11: Frustration towards others	0.64%	0.21%	2.05%	4.65%	8.98%	14.5%
12: Distrust in media and government	2.70%	2.70%	2.59%	6.27%	5.84%	11.8%
13: Does not need support	2.05%	0.64%	0.64%	5.62%	0.21%	2.98%
−999: Scored neutrally as difficult to interpret	11.1%	3.89%	1.73%	8.11%	14.3%	
−99: Missing	22.5%	22.7%	24.7%	17.9%	25%	

### Covid-19 restrictions, such as social distancing and travel restrictions, have negatively impacted people’s livelihoods

Our first question was on how people have been affected by the Covid-19 pandemic. A minority of participants mentioned no changes in lifestyle, behaviour or thinking for the better (21.8%) or for the worse (17.3%), the majority reported positive (78.2%) or negative changes (82.7%) in motivation, work, studies and difficulties in accessing services or support due to the impact of Covid-19 policies. Many participants reported a general lack of motivation and concentration due to isolation and having to adapt to a changed balance in the environment including spending more time at home. Many also reported being more negative when it came to feelings about the future, ranging from ‘feeling optimistic about the future to ambivalent at best’. This sentiment often presented alongside a change in work environment or work–life balance, and even annoyance in the mundane space and environment where the line for work-and-play is blurred, ‘getting so bored working from home’.

Another theme centred on how the Covid-19 pandemic impacted participants’ finances, work and studies. Many participants spoke of how changes to the work environment and workload have negatively or positively impacted their livelihoods. Participants reported widespread issues including worries about long-term job security, such as worries about ‘teaching contract[s] not being extended’, and the impact of drastic increases in workload since working from home (e.g., ‘work–life balance has decreased significantly’; ‘My workload has increased a lot last year and I have job insecurities’; ‘My work has been moved primarily online … which has resulted in my workload increasing by at least 50% in terms of effort and time’). For some participants, these issues were further compounded by pre-existing financial struggles, and they reported a desperate need for a steady cash flow just to get by. The impact of an increase in workload and work-related stressors further impacted participants’ relationships (e.g., wanting ‘a workload that isn’t crippling so I can spend more time with my son’). Furthermore, participants also voiced their frustrations about not being able to see family due to tighter restrictions and, for some, not being able to grieve over the loss of their loved ones. Whilst staying socially connected with others has been proven difficult during Covid-19, the responses further highlighted the impact of lockdown restrictions on people’s access to services such as mental health support, including the pandemic being a stimulus to starting therapy or counselling sessions for those who can afford it (e.g., ‘I started online therapy … knowing that this was going to be a rough ride’; ‘I learned how to deal with trauma memories … [after] attempted suicide in February’; ‘starting to attend trauma therapy’).

### People’s attitudes toward themselves and others have changed for the better and worse

The pandemic prompted significant changes in people’s outlook on life and this theme appears to be the most prominent (37.7% of all responses). This included significant changes in participants’ attitudes toward others. Firstly, participants reported less trust towards governments due to their response to the pandemic, as shown in policymaking. Some participants described their government as ‘selfish’, ‘corrupt’ or ‘self-serving’. Dissatisfaction with governments’ Covid-19 response also included ‘vaccine role out’, ‘financial cuts’ and not being able to ‘keep infections under control’. There was an overarching sense that what participants wanted was ‘a government that is focused on supporting people rather than pandering to their financial backers’, and for governments to focus on implementing evidence-based support systems to local communities.

Secondly, participants had reported feeling ‘angry’, ‘frustrated’ and ‘depressed’ about the spreading of Covid-19 misinformation ‘shared… [on] social media’. Thirdly, frustration towards others over differences in opinion on how strongly one should adhere to Covid-19 policies (e.g., social distancing or getting vaccinated) was observed. Participants commented on how ‘lots of people don’t care about others’, and how the pandemic has shown them just ‘how selfish some people are’ and how some people are ‘unwilling to make sacrifices to protect other(s)’. Lastly, mixed impressions towards friends and family were reported. While some participants were ‘more appreciative of their friends and family’, others commented they have learned ‘who their real friends are’, suggesting that reduced social contact with loved ones has prompted periods of introspection and reassessment. Emotions were mixed for some participants who moved back in with their families to weather out the pandemic, including feeling ‘more irritable’, ‘more frustrated’, ‘more thankful’ and that ‘talking [to them] helped them cope and validate their feelings’.

Thirdly, the Covid-19 pandemic has also changed participants’ attitudes toward themselves, providing ‘more time to understand their jobs’, ‘find new opportunities after they finish [their] studies’, and made them ‘excited’ to ‘reconnect with friends and family’. Some expressed how the pandemic has prompted them to rethink their current priorities in life, bringing about ‘significant changes in terms of their lifestyle, behaviour and thinking’. Others have found the pandemic to be a transformative experience of ‘learning’, ‘realisation’ and ‘rediscovery’, afforded in the changed ‘single’ environment in which they are forced to dwell, learn, and work in, prompting self-reflection on the areas of their life, which perhaps is not unlikely pre-pandemic times when an individual may have had to actively seek refuge and solace in their own private space due to too much social interactions with others. During the pandemic, changes in an individual’s outlook on life and on themselves have therefore encouraged many participants to be more motivated and optimistic for their future.

### People’s mental and physical health have been primarily negatively impacted by the Covid-19 pandemic with some positive impacts

Many participants reported how their mental health was negatively impacted by the pandemic. Participants who were living alone during lockdown largely reported feeling lonely and missing social contact from their loved ones. Participants further expressed feeling ‘more anxious’, ‘constant anxiety’ or worried about ‘being around other people’, and some expressed that they would rather be on their own to minimise the risk of contracting Covid-19. Participants reported mixed success in how they have coped with Covid-19, with some feeling ‘more resilient’ and others that their mental health was the worst it has ever been (e.g., ‘all time low’; ‘rock bottom’).

Participants’ physical health was also negatively impacted by the pandemic. Unsurprisingly, many participants spoke about reduced physical exercise and social activities with others, in line with the Covid-19 restrictions, which have prevented people from ‘visit(ing) friends and family abroad’ and has contributed to more sedentary behaviours such as staying indoors and at home for longer periods of time. Some participants recounted poorer physical health due to increases in alcohol consumption (e.g., ‘drinking more alcohol’; ‘worse alcohol intake’; ‘drink more, put on weight’), drug usage (e.g., ‘doing cocaine again’; ‘relapsed into smoking/vaping’) or ‘addiction to social media’. For other participants, such behaviours resulted in stronger ‘reluctance to leave home’ for exercise or social contact. These experiences are perhaps not unlikely the experiences of individuals who even pre-pandemic times have had to live with health vulnerabilities (e.g., asthma, ageing populations with reduced access outdoors due to high pollution levels, individuals with reduced mobility) and restricted environments (e.g., curfew, parole, elderly home).

Even so, some participants also described an increase in coping behaviours. Examples included making more effort to stay in touch with friends and family virtually (e.g., ‘increased socialisation through social means’) and practicing meditation and mindfulness (e.g., ‘sustained a meditation regime’; ‘meditation, reflecting, prayer’).

Overall, while the impacts of Covid-19 were largely negative, certain individuals were finding ways to cope.

### Group differences on the Covid-19 experience

Many people expressed the need for more support as part of the post-Covid-19 recovery. Support that extends beyond solely financial support was preferrable (e.g., ‘better access to physical and mental health support and if necessary, treatment, would be of huge impact to me and my family’). To identify potential group differences in the frequency of codes, independent t-tests were conducted on gender, country, age and income groups. There were significant group differences in in participants aged above and below 38 years for all codes except for code 9 (Loss/bereavement). No group differences were found for country (codes: 1/Mental health; 5-/Loss of motivation and optimism; 7/Covid-19 policies; 8/Access to services and support: 9/Loss, bereavement; and 13/No support needed), income (codes: 1/Mental health; 2/Outlook on life, self; 4-/Sedentary behaviours; 5-/Loss of motivation and optimism; 6/Finances, work, studies; 7/Covid-19 policies; 8/Access to services and support; 9/Loss, bereavement; 10/Virtual living, virtual events; 11/Frustration towards others; 13/No support needed; and −999/Neutral answers), and gender (codes: 3/Loved ones; 4-/Sedentary behaviours; 5-/Loss of motivation and optimism; 7/Covid-19 policies; 8/Access to services and support; 9/Loss, bereavement; 10/Virtual living, virtual events; 11/Frustration towards others; 12/Distrust towards government and media; and 13/No support needed) accounting for sample size differences in groups (see Appendix 5 for visual Venn diagrams). Of the codes where there were significant group differences in the frequency of the codes, further analysis suggests that responses do not differ qualitatively in the support they wanted. Below are some example quotes from different groups.

Out of all the countries, Italy and UK participants reported significantly more Covid-19 induced changes in their outlook towards life and themselves (see Table 5). For example, one participant from Italy describes how the pandemic has negatively impacted their perception of the world: ‘*My confidence in humanity has dropped extremely. A pandemic could be the common enemy, that ploy that humanity needed to act and interact as one people. Instead, EVERY individual has thought of their own interests.’ [La mia fiducia nell’umanità è estremamente calata. Una pandemia poteva essere il nemi o comune, quell’escamotage di cui l’umanità aveva bisogno per agire ed interagire come un sol popolo. Invece OGNI singolo ha pensato ad i propri interessi*.] This is consistent with the fact that most of our respondents at time point 3 were from the UK and Italy. Participants from all countries reported loved ones having a significant impact on their well-being during Covid-19. In particular, ‘not being able to see family or friends’ or trying to ‘keep in touch with long-distance friends and family more often’ was challenging. Participants further expressed guilt and concern about their loved ones when they were ‘not able to travel to see parents in their home country’ and ‘friends desperately needing money for living expenses, therapy’. However, compared to the UK, participants from the US reported engaging in more sedentary behaviours and lifestyle during Covid-19, acknowledging that they were lacking ‘a variety with activities to do and places to go’, lockdowns have them ‘feeling restrained’ and that they are ‘not exercising or as active as once was’. Similar sentiments were reported in Italy as well: ‘*My cardinal sin is laziness, and the lockdown has only allowed me to indulge in sloth’.* [*Il mio peccato capitale è la pigrizia e il lockdown mi ha solo permesso di indulgere nell’accidia*.]

**Table 5.** Analysis of code frequencies by country

Code	Countries	Significance
1: Mental health (perceptions, cognitions, feelings)	UK = US	*z* = 0.6, *P* = 0.528
	Italy = Greece	*z* = 0.5, *P* = 0.617
	UK = Greece	*z* = −0.0, *P* = 0.0968
	US = Italy	*z* = −0.2, *P* = 0.852
	UK = Italy	*z* = –0.07, *P* = 0.447
	US = Greece	*z* = 0.3, *P* = 0.726
2: Outlook on self/life	UK = US	*z* = 1.7, *P* = 0.071
	Italy > Greece	*z* = 2.7, *P* = 0.005
	UK > Greece	*z* = 3.4, *P* < 0.0006
	US = Italy	*z* = −1.7, *P* = 0.081
	UK = Italy	*z* = −0.08, *P* = 0.400
	US = Greece	*z* = 1.4, *P* = 0.138
3: Loved ones (friends, family)	UK = US	*z* = −0.1, *P* = 0.865
	Italy = Greece	*z* = 0.3, *P* = 0.748
	UK = Greece	*z* = −1.5, *P* = 0.128
	US = Italy	*z* = −1.6, *P* = 0.096
	UK > Italy	*z* = −1.9, *P* = 0.045
	US = Greece	*z* = −1.2, *P* = 0.207
4-: Sedentary behaviours (inactive, decrease in behaviours)	UK = USA	*z* = 0.3, *P* = 0.741
	Italy = Greece	*z* = 1.9, *P* = 0.057
	UK = Greece	*z* = −0.3, *P* = 0.696
	US > Italy	*z* = −2.7, *P* = 0.006
	UK > Italy	*z* = −2.8, *P* = 0.003
	US = Greece	*z* = −0.5, *P* = 0.582
4+: Self-improvement (active, increase in behaviours)	All countries	*z* = NaN, *P* < 0.001
5+: Motivation/optimism about the future	UK > US	*z* = −2.8, *P* = 0.004
	Italy = Greece	*z* = −0.1, *P* = 0.849
	UK = Greece	*z* = −0.09, *P* = 0.337
	US = Italy	*z* = 1.0, *P* = 0.289
	UK = Italy	*z* = −0.07, *P* =.477
	US = Greece	*z* = 0.8, *P* = 0.412
5-: Loss of motivation/optimism about the future	UK = US	*z* = 1.3, *P* = 0.177
	Italy = Greece	*z* = 0.9, *P* = 0.352
	UK = Greece	*z* = 2.3, *P* = 0.016
	US = Italy	*z* = 0.1, *P* = 0.912
	UK = Italy	*z* = 1.1, *P* = 0.238
	US = Greece	*z* = 1.1, *P* = 0.246
6: Finances/work/studies	UK = US	*z* = 1.3, *P* = 0.177
	Italy = Greece	*z* = 0.9, *P* = 0.352
	UK > Greece	*z* = 2.3, *P* = 0.016
	US = Italy	*z* = 0.1, *P* = 0.912
	UK = Italy	*z* = 1.1, *P* = 0.238
	US = Greece	*z* = 1.1, *P* = 0.246
7: Covid-19 policies	UK = US	*z* = 0.7, *P* = 0.718
	Italy = Greece	*z* = −0.1, *P* = 0.857
	UK = Greece	*z* = −0.2, *P* = 0.818
	US = Italy	*z* = 0.2, *P =* 0.810
	UK = Italy	*z* = 0.0, *P* = 0.992
	US = Greece	*z* = −0.0, *P* = 0.984
8: Access to support/services	UK = US	*z* = 0.3, *P* = 0.726
	Italy = Greece	*z* = 1.2, *P* = 0.193
	UK = Greece	*z* = 1.0, *P* = 0.289
	US = Italy	*z* = −0.7, *P* = 0.435
	UK = Italy	*z* = −0.6, *P* = 0.522
	US = Greece	*z* = 0.7, *P* = 0.465
9: Loss/bereavement	All countries	*z* = NaN, *P* > 0.005
10: Virtual living/virtual events	UK > US	*z* = −3.7, *P* < 0.001
	Italy = Greece	*z* = −0.4, *P* = 0.689
	UK = Greece	*z* = −0.4, *P* = 0.667
	US = Italy	*z* = 1.3, *P* = 0.161
	UK = Italy	*z* = −1.0, *P* = 0.298
	US = Greece	*z* = 1.7, *P* = 0.078
11: Frustration towards others	UK > US	*z* = 1.9, *P* = 0.046
	Italy = Greece	*z* = −0.2, *P* = 0.794
	UK = Greece	*z* = 0.8, *P* = 0.417
	US = Italy	*z* = −0.9, *P* = 0.327
	UK = Italy	*z* = 0.5, *P* = 0.603
	US = Greece	*z* = −0.6, *P* = 0.524
12: Distrust in media and government	UK > US	*z* = 2.8, *P* = 0.003
	Italy = Greece	*z* = 1.6, *P* = 0.105
	UK = Greece	*z* = 1.3, *P* = 0.170
	US > Italy	*z* = −2.6, *P* = 0.008
	UK = Italy	*z* = −0.7, *P* = 0.447
	US = Greece	*z* = −0.6, *P* = 0.496
13: No support needed	UK = US	*z* = −0.1, *P* = 0.912
	Italy = Greece	*z* = 1.0, *P* = 0.293
	UK = Greece	*z* = −1.3, *P* = 0.167
	US = Italy	*z* = 0.1, *P* = 0.904
	UK = Italy	*z* = 0.0, *P* = 0.960
	US = Greece	*z* = −1.0, *P* = 0.274
−99: Missing	All countries	*z* = *NaN*, *P* < 0.001
−999: Scored neutrally as difficult to interpret	UK > US	*z* = −2.5, *P* = 0.012
	Italy = Greece	*z* = −1.0, *P* = 0.280
	UK = Greece	*z* = 0.7, *P* = 0.483
	US = Italy	*z* = 1.0, *P* = 0.317
	UK = Italy	*z* = −0.7, *P* = 0.453
	US > Greece	*z* = 2.1, *P* = 0.031

NaN = very small number.

Participants from the UK reported having the most optimism for the future, with many participants expressing their excitement for ‘things to return to normal’, ‘moving freely in society’ and ‘travelling abroad’. In addition, participants from the UK spoke significantly more than those in other countries about their experiences with work, studies and financial disruptions and silver linings that arose from the pandemic. There were mixed feelings about ‘working from home’ but employees wanted ‘more flexibility’ when returning to the work force.

Participants found technology to be a saving grace and a hindrance to their work and social lives, with participants from the UK reporting significantly more that ‘online support groups’, ‘online workshops’ and ‘Zoom calls’ were the most popular way for participants to stay connected with friends and family and to work with colleagues from home. Some participants spoke about ‘online lectures’ and ‘online teaching’. In particular, participants from Greece found that ‘online lectures were less effective compared to those with physical presence’, but overall there was a sense that Covid-19 enabled participants from all countries to get ‘used to online teaching and learning’.

Participants from the US and the UK reported significantly more frustration towards others, ‘people are selfish’ and that Covid-19 has revealed ‘how unkind and insensitive most people are’ and in some cases, deteriorating relationships with loved ones, led people having to move houses. Frustration towards others also extend to governments and news outlets. Participants’ sentiments towards the US and UK administration have been emphasised to be worsening: ‘the pandemic has taught me just how little this government cares about the everyday person and important issues’. Participants expressed their ‘trust in government has deteriorated’ and that they ‘feel much worse about the state of our country’. The media was found to be a key source of stress for all participants, but particularly in the US and the UK where participants expressed that they ‘hate reading the news because it always makes me sad’ and that sometimes the ‘anger of the state of the world would consume me’.

In terms of gender, although there were significant differences in the frequency of some codes between male and female participants (see Table 6), participant’s responses were not qualitatively different. Both men and women spoke about changes in lifestyle as a result of Covid-19 where they had to remind themselves ‘not rushing around and trying to do too much’ or to ‘prioritise myself more and stick to my boundaries. Exercise more. Eat better’. Some participants gained insight about themselves even though they did not think they would cope, ‘that I am comfortable with my own company’ and that it was possible to ‘set short term goals and take my health into my own hands’.

**Table 6.** Analysis of code frequencies by gender

Code	Significance
1: Mental health (perceptions, cognitions, feelings)	F > M
	*z* = −4.1, *P* < 0.001
2: Outlook on self/life	F > M
	*z* = 2.4, *P* = 0.012
3: Loved ones (friends, families)	F > M
	*z* = 1.1, *P* = 0.246
4+: Self-improvement (active, increase in behaviours)	F > M
	*z* = NaN, *P* < 0.001
4-: Sedentary behaviours (inactive, decrease in behaviours)	F > M
	*z* = −0.1 = 2, *P* = 0.155
5+: Motivation/optimism about the future	F > M
	*z* = 1.9, *P* = 0.048
5-: Loss of motivation/optimism about the future	F > M
	*z* = 0.6, *P* = 0.496
6: Virtual living/virtual events	F > M
	*z* = 2.5, *P* = 0.120
7: Covid-19 policies	F > M
	*z* = −1.8, *P* = 0.062
8: Access to support/services	F > M
	*z* = 1.0, *P* = 0.317
9: Loss/bereavement	F > M
	*z* = −0.7, *P* = 0.435
10: Virtual living, virtual events	F > M
	*z* = 0.8, *P* = 0.417
11: Frustration towards others	F > M
	*z* = 0.4, *P* = 0.652
12: Distrust towards government and media	F > M
	*z* = 0.7, *P* = 0.435
13: No support needed	F = M
	*z* = 1.1, *P* = 0.238
−99: Missing	F > M
	*z* = NaN, *P* < 0.001
−999: Scored neutrally as difficult to interpret	F > M
	*z* = 2.3, *P* = 0.020

When comparing groups earning more or less than £40,000 per year to see whether they cared about different things, although there were significant differences in the frequency of codes for some codes, their responses were not qualitatively different (see Table 7). Many respondents spoke about having, ‘little faith in the process of government in getting vaccines to people or managing lockdowns or crisis situations’. Some spoke about the need for the government to be held responsible for their poor handling of the pandemic, ‘for the government to be held to account for their horrific failings to relieve the sense of injustice I feel’. Still, others spoke about their resilience and lessons learned from the pandemic, ‘Yes, that I can cope with a sedentary lifestyle reasonably well; and those others are more prone to stress than I thought’, missing interactions with colleagues, ‘Not seeing coworkers is not great. I miss them’, and friends, ‘I miss seeing people more than I thought I would’. No real differences in content were observed between groups.

**Table 7.** Analysis of code frequencies between participants with a higher income of £40,000 per year (I_1_) and participants with a lower income of £40,000 per year (I_2_)

Code	Significance
1: Mental health (cognitions, feelings, perceptions)	I_1_ = I_2_
	*z* = 0.6, *P* = 0.483
2: Outlook on life/self	I_1_ = I_2_
	*z* = −1.7, *P* = 0.081
3: Loved ones (friends and family)	I_1_ > I_2_
	*Z* = −2.5, *P* = 0.012
4+: Self-improvement (active, increase in behaviors)	I_1_ < I_2_
	*z* = NaN, *P* < 0.001
4-: Sedentary behaviours (inactive, decrease in behaviours)	I_1_ = I_2_
	*z* = −0.4, *P* = 0.681
5+: Motivation/optimism about the future	I_1_ < I_2_
	*z* = −1.9, *P* = 0.047
5-: Loss of motivation/optimism about the future	I_1_ = I_2_
	*z* = −1.7, *P* = 0.089
6: Finances/work/studies	I_1_ = I_2_
	*z* = −0.1, *P* = 0.920
7: Covid-19 policies	I_1_ = I_2_
	*z* = 0.0, *P* = 0.960
8: Access to support and services	I_1_ = I_2_
	*Z* = −0.2, *P* = 0.841
9: Loss/bereavement	I_1_ = I_2_
	*z* = 0.0, *P* = 0.960
10: Virtual living/virtual events	I_1_ = I_2_
	*z* = 1.5, *P* = 0.128
11: Frustration towards others	I_1_ = I_2_
	*z* = 0.3, *P* = 0.741
12: Distrust in media and government	I_1_ < I_2_
	*z* = −2.7, *P* = 0.005
13: No support needed	I_1_ < I_2_
	*z* = 0.8, *P* = 0.417
−99: Missing	I_1_ > I_2_
	*z* = NaN, *P* < 0.001
−999: Scored neutrally as difficult to interpret	I_1_ = I_2_
	*z* = 1.7, *P* < 0.075

### Group differences on support

When asked what type of support people wanted, there were no particularly stark contrasts in their view of support across countries, age, gender and income.

Both men and women spoke about the return to pre-pandemic environmental conditions in which they lived with the possibility and hope that Covid-19 vaccines can bring about an ending to the pandemic, ‘vaccines, money, ability to travel again (international family)’ and how they would want travel restrictions to be lifted to allow everyone to reunite with distant families again, ‘permission to travel to see family, efficient vaccines’. Participants aged above and below 38 both spoke about the pandemic having a toll on their mental health, some feeling ‘an all time low’ and others feeling ‘more resilient and stronger than they think’, as well as ‘missing friends’, ‘family’ and ‘colleagues’. Participants from various income groups spoke about feeling frustrated that they ‘can’t host large gatherings’ and working in Covid-19 times has been ‘more challenging’, whether working from home or in face-to-face professions. Income was not a predictor of whether participants needed significantly different kinds of support, as some participants regardless of income reported ‘nothing comes to mind’, ‘nothing’ or ‘we’re doing fine’.

### Other relevant themes

From our data, we also identified a small but specific vulnerable population who spoke about having suffered disproportionately throughout the pandemic. These populations include single parents who described having ‘struggle[s] with childcare, [and had] started therapy [and even] started therapy for [their] kids’. Another group identified is individuals trapped in unstable and unsafe relationships, where some participants reported needing to move out of their homes due to relationship conflict and breakdown (e.g., ‘I need to buy a house real quick so I can move out … I have no support from anyone’).

## Discussion

### Main findings

The aim of this study was to explore the impact of the Covid-19 pandemic on people’s experiences, perspectives and livelihoods. To the best of our knowledge, this is the first study to examine a range of socioeconomic, behavioural and mental health impacts of the Covid-19 pandemic across countries in a large sample of over 900 participants. Each of our study findings are discussed in turn.

#### Theme 1: Covid-19 restrictions such as social distancing and travel restrictions, have negatively impacted people’s livelihoods

With regard to our first hypothesis, our study has uncovered three main themes comprised of 13 codes capturing the wide ranging positive and negative impacts of the pandemic on different populations. It is clear that Covid-19 lockdown restrictions have led to decreased motivation and concentration, increased workload and worries relating to long-term job security, and distrust towards government policy and action – similar to experiences reported by individuals working in healthcare [11,12,27], young adults in school [18,19] and education [13]. Restrictions also preceded the increased use of mental health services – for those who were able to access free counselling or those who had the financial capital to afford private services – yet highlighting those who were not able to have continued access or afford mental health and healthcare support during the pandemic. These findings were consistent with past studies uncovering difficulties in mental health access [26] from those with existing mental health conditions [14] and families with young children and children with special education needs [15–17,19].

Covid-19 restrictions, such as social distancing and travel restrictions, significantly contributed to a negative impact on livelihoods across the world. Many participants reported feeling despondent and reduced motivation and concentration from needing to spend more time at home. Finances, work and studies (Code 6) was particularly prevalent, with participants reporting struggles with long-term job security and stable funding, which is consistent with past studies. This has further implications for their ability to afford healthcare and essentials, as shown in past studies [19], identifying a potential vulnerable group that deserves further support and attention.

#### Theme 2: People’s attitudes towards themselves and others have changed for the better and worse

Second, the pandemic has also caused changes in self-perception. Many reported introspective self-discoveries, such as knowing more about themselves. Often times, this was followed by greater optimism and motivation for the future that indicated significant personal growth, a more optimistic outlook when compared to studies of older age groups [22]. It was clear that greater time spent in isolation prompted episodes of self-revelation and discovery for many. We also received many responses indicating distrust in others due to the apparent lack of responsible action taken, which has also been found to be associated with poorer mental health [20] and adoption of health behaviours [31]. Varying attitudes towards how governments across the world have supported or unsupported their citizen’s recovery from the pandemic were also observed. Some participants expressed frustration and having a lowered or lack of trust in their government.

#### Theme 3: People’s mental and physical health have been primarily negatively impacted by the Covid-19 pandemic with some positive impacts

Third, people reported a toll on health – both mentally and physically. While the minority – 2.91% – reported minimal distress, coped adequately and/or reported improved mental health, an alarming number of responses illustrated deterioration of mental health and an inability to cope with significant life stressors, 66.85%. This was especially for those who lived alone and individuals who were already battling with pre-existing mental health difficulties, consistent with previous qualitative studies [23,26]. We know from past studies that mental health symptoms fluctuate throughout the pandemic lockdown periods for both adults and young children, thus more mental health support should be deployed for especially strict lockdown periods [2,3,17]. For this group, it seems that changes in lifestyle habits (e.g., social isolation, productivity and habits) became stressors for the onset of mental health problems including self-harm behaviours to ruminative thinking and symptoms of anxiety and depression, which has also been evidenced in past studies [20]. Our participants also spoke of worsening physical health, in the form of increased substance abuse and addictions (e.g., alcohol, food, social media) – a phenomenon observed in other studies showing increased risk for overdose during the pandemic [32] – and the reluctance to leave home to engage in social or physical activity [33,34]. However, 20.6% of participants also reported positive physical health outcomes due to an increased effort to exercise, practicing mindfulness and meditation or reducing their substance use.

#### Group comparisons on inequalities

By comparing data across country and income groups, significant differences in code frequencies were found for some country-pairs (in the case of UK–US, UK–Italy and UK–Greece) as well as between gender. No significant differences were found for age and income levels. In comparison to countries such as China, South Korea and Singapore who maintained a proactive approach by tracking and isolating close contacts to identify and manage cases, the UK and the US are said to have responded with mitigation strategies that focused on treating severe cases and cases with pre-existing health concerns [35]. A more detailed examination of Covid-19 strategies between the US and the UK is needed. Similarly, previous literature reviews have identified similarities in mitigation and suppression strategies adopted by Italy and the UK [36]. The current lack of evidence regarding the types of Covid-19 control strategies implemented has meant that different countries have adopted varying mitigation and suppression strategies. As it stands, much ambiguity surrounds the cogency of preventative measures such as lockdowns, work policies, quarantining, social gathering policies, etc. The types of strategies implemented vary depending on socio-cultural, technological or political factors.

Still, the pandemic’s effect on its citizens has been mitigated or exacerbated depending on their country’s Covid-19 restrictions. The UK Government’s Covid-19 response strategy in the spring of 2021 allowed the opening of non-essential retail (e.g., hairdressers) and public buildings such as libraries, community centres, indoor leisure facilities and outdoor attraction and hospitality venues. From 21 June 2021, all legal limits on social contact were removed. In contrast, European nations such as Greece and Italy adopted the use of ‘vaccine passports’ as a means to attract tourists for the summer of 2021. In addition, the Presidents of the European Parliament, the European Union (EU) Council and the European Commission made the EU Digital Covid Certificate official, which marked the end of Europeans’ travel restrictions within their own and neighbouring EU countries. Alternatively, in the US, Covid-19 restrictions were highly variable and dependent on individual state and county legislation, with ten states having never issued a ‘stay at home’ order. Out of 50 states, 23 did not issue legislation on the use of face coverings but all 50 states closed their schools for the remainder of the 2021 spring/early summer term.

*Other relevant themes.* A recurring, yet unexpected theme in the responses revealed that many participants had used the questionnaire as an avenue to air out their worries or concerns and to rant, almost as a form of catharsis (see Appendix 6 for examples). In these ruminations, participants often identified points of ‘realisation’ and recounted their reactions to situations and identified their resultant thoughts or emotions. For example, the longest recorded response was 697 words long (see ID 235 in Appendix 6) and dictated a response about the types of support this participant and their family may need in the next 6 months. The fact that a considerable number of participants took the time to exhaustively report their thoughts and feelings in the questionnaire highlights how many felt emotionally overwhelmed at the point of data collection. Due to the questionnaire being anonymous, perhaps participants felt more comfortable recounting exceedingly detailed information about their personal lives and experiences. Such detailed responses emphasise the strong desire for people to be heard in periods of crisis, especially having been isolated from social life for such an unprecedented period.

The questionnaire was successful in capturing the experiences from individuals in various populations considered vulnerable and those not so much affected. These responses presented a sharp contrast to those who reported not needing much support for post-pandemic recovery, highlighting the vast demographic disparities that have arisen or have been exacerbated by Covid-19. As Covid-19 restrictions have clearly affected individuals disproportionately, future research should explore the pandemic’s unique impact on vulnerable populations, including single mothers and individuals who feel as though they are in an unstable or unsafe relationship, families seeking psychological support, and identify possible avenues for support for those who will need more than 6 months to recover from the pandemic.

#### What support do people need?

While many respondents reported that they did not need any form of support (2.41%), 24% of participants expressed that changes in work-related practices would help (e.g., ‘[I] need my work to be understanding with childcare’; ‘reduced workload’). In addition, 22% of participants expressed the need for either access to or continued ‘mental health support’ or ‘therapy’ (e.g., ‘counselling or other mental health services to deal with the trauma of the past year’; ‘I would appreciate … better access to mental health support because I am not a citizen, I do not qualify for mental health care’). Overall, there was a clear demand for support both at the individual (mental health, finances) and community level (workplace, local infrastructure). Of those who wanted more support, 91% of participants expressed that they needed more support for their post-Covid-19 recovery – support that extends beyond financial support. And although our responses reveal no ‘qualitative’ differences between groups, people with existing conditions and fewer resources are likely to have experienced the impacts of the pandemic even more that may not necessarily be fully captured in our open-ended questions.

The findings from this study emphasises that global leaders and governments should prioritise their citizens’ mental health, social relationships, and access to services, and that more funding and resources should be allocated to key organisations that serve their citizens.

## Strengths and limitations

This study is not without limitations. First, the lack of pre-pandemic data on participants’ situation and health limited our ability to assess real change and impact beyond self-reported data, which will ultimately have a certain level of bias. Thus, future studies triangulating participant data across official clinical health database and self-report data will overcome this limitation. Second, an open-ended survey from a global convenient sample does not allow follow-up elaborations and the translations of non-English responses, although fairly accurate, are both taken at face value and may not capture the nuances that some participants may have intended. Third, participants from different countries have experienced varying levels of Covid-19 restrictions and so the collected responses may reflect only those who have been most impacted in countries with strict lockdowns rather than less severe lockdowns (e.g., the UK had national lockdowns whilst most Asian countries did not have full national lockdowns). Hence, future qualitative studies from specific countries can help address this limitation.

Despite these limitations, this study has several noteworthy strengths. First, a key strength of this study is the large, cross-country sample representing a range of ages and socio-economic statues that has allowed testing for group differences. Second, thematic analysis was conducted blind to participants’ demographic data, which minimised researcher bias on the impact of the pandemic, increasing the validity of our interpretations. Third and finally, the strongest aspects of this study were the long responses from the study participants. While participants were not paid (only entered into a prize raffle), the detailed responses reflected a highly motivated and willing group of participants who wished to share their insights with the study team.

## Conclusion

The Covid-19 pandemic has fundamentally altered the environment in which people lived as a result of a range of controlled measures that are still being felt globally in different locally communities and globally. This study shows how this changed individuals’ perceptions of themselves and the world, whilst also highlighting the substantial inequalities in support for the most vulnerable in times of crisis. This pandemic is a wake-up call for communities around the world to be better prepared for future pandemics to come. Whilst popular media coverage focuses on Covid-19 pandemic’s impact on the global economy, our study findings contribute to the growing literature advocating for more consideration for the impact on individual’s perceptions, behaviours and relationships. Lockdowns have clearly taken a toll on everyone, but particularly for vulnerable groups who find themselves isolating with limited support during these challenging times. Non-profits and third sectors working tirelessly to support vulnerable groups need additional funding to provide adequate support, not funding cuts. Local communities and authorities with potential to provide targeted support are lifelines to the population that can help bridge citizens’ growing distrust and dissatisfaction toward governments and their pandemic policies. The time is now: Ahead of other pandemics to come, we need to *mend relationships* between individuals and governments globally, *build resilient systems* and motivate world leaders to work together to *recover stronger* from this pandemic.

## Data Availability

The datasets generated during and/or analysed during the current study are available in the repository: https://osf.io/fe8q7.

## Appendix 1

**Table A1.** Responses by country

Countries		n	%
Argentina		1	0.1%
Australia		16	1.7%
Austria		1	0.1%
Barbados		1	0.1%
Belgium		1	0.1%
Brunei Darussalam		1	0.1%
Bulgaria		1	0.1%
Canada		**24**	2.6%
China		**19**	2.1%
Colombia		3	0.3%
Cyprus		2	0.2%
Finland		2	0.2%
France		5	0.5%
Gambia		1	0.1%
Germany		22	2.4%
Greece		**51**	5.5%
Hong Kong (S.A.R.)		**28**	3.0%
India		11	1.2%
Indonesia		8	0.9%
Ireland		2	0.2%
Israel		3	0.3%
Italy		**58**	6.3%
Jamaica		1	0.1%
Japan		4	0.4%
Lebanon		2	0.2%
Luxembourg		2	0.2%
Malaysia		5	0.5%
Malta		2	0.2%
Mexico		4	0.4%
Netherlands		7	0.8%
New Zealand		7	0.8%
Norway		1	0.1%
Pakistan		1	0.1%
Philippines		6	0.6%
Poland		4	0.4%
Portugal		4	0.4%
Qatar		2	0.2%
Republic of Moldova		1	0.1%
Romania		1	0.1%
Rwanda		1	0.1%
Saudi Arabia		1	0.1%
Singapore		13	1.4%
South Africa		1	0.1%
Spain		2	0.2%
Sweden		7	0.8%
Switzerland		4	0.4%
Turkey		1	0.1%
United Arab Emirates		2	0.2%
United Kingdom of Great Britain and Northern Ireland		**443**	47.8%
United States of America		**107**	11.6%
Missing	−9	29	3.1%

Note: **Bold** values indicate countries with a large enough sample size to be included in the Venn diagrams.

**Table A2.** Housing status

		N	%
En-suite (renting)		29	3.1%
Single bedroom flat (renting)		87	9.4%
Double bedroom flat (renting)		134	14.5%
Room in shared house (renting)		75	8.1%
House (renting)		87	9.4%
En-suite (owned)		7	0.8%
Single bedroom flat (owned)		13	1.4%
Double bedroom flat (owned)		79	8.5%
Room in shared house (owned)		21	2.3%
House (owned)		316	34.1%
Other		41	4.4%
Missing	−99	4	0.4%
	−9	33	3.6%

## Appendix 2

**Table A3.** Number of missing data cells (−99) and cells where answers were scored neutrally as difficult to interpret (−999) by question

Question number	Frequency of (−99)	Frequency of (−999)
Q52	208	102
Q71	208	37
Q72	229	15
Q73	165	75
Q74	230	133

## Appendix 3

### Achieving inter-rater

#### Calibration meeting 1

In the first calibration meeting, the team found that code 2 (‘outlook on life’) was applied inconsistently. Its definition was subsequently revised to encompass only introspective responses. We also highlighted that codes 4+ (‘doing more activities’) and 4- (‘sedentary behaviours’) were assigned to responses which were behavioural in nature. During this meeting, 37% of responses were blank, 29% of responses had a code match and 71% of responses did not have a code match. These responses were discussed, and discrepancies were addressed. In the second calibration meeting, 22 cells were blank, 64% of responses had a code match and 36% of responses did not have a code match. These responses were discussed, and discrepancies were addressed.

#### Calibration meeting 2

After the second calibration meeting, the team came to a consensus to code responses mentioning an ‘increase in social distancing’ or ‘keep(ing) distance’ as (7,4-). While it was an increase in behaviour, the response was treated as a decrease in socialisation. It was also noted that any mention of money or finances was to be coded as 6. Responses that mentioned ‘work from home’ were also to be coded as 6. Responses that mentioned ‘friends or family’ were to be coded as 3. Responses that mentioned ‘studies’ were also to be coded as 6. Two more codes were added to the coding scheme: ‘−999’ (to denote responses scored neutrally as difficult to interpret e.g., ‘not really’, ‘no’, ‘yes’ with no further explanation) and ‘−99’ (for blank responses). The team achieved an IRR of 75% by the third calibration meeting (see Table 2 for final code scheme), where the team evaluated 31 cases, of which 11 were blank and 5 were discussed.

#### Calibration meeting 3

KM and KL reviewed the codes for responses to Q52, KL reviewed codes for responses 1–463 and KM reviewed responses 464–927. KL added an additional tab to the coding Excel spreadsheet called ‘Consensus’ in which matching code cells were identified by a green ‘Match’ label and non-matching code cells were identified by a red ‘No Match’ label. IRR was significantly increased by ensuring all blank cells were coded as ‘−99’ and the order of matching codes was the same in both coder’s columns. For the ‘No Match’ cases, an alternative code set was proposed and highlighted in blue. After a team meeting on 20th October 2021, it was decided that the alternative code sets would be used for the ‘No Match’ cases. By the end of this process, IRR for the responses to Q52 was 81.2%.

## Appendix 4

### Quotations from the results section

*Covid-19 restrictions such as social distancing and travel restrictions, have negatively impacted people’s livelihoods*

**ID 804:** *‘Feeling guilty about being less productive than usual, not being able to see family (abroad) or friends, not being able to engage with activities outside of the home, particularly the social kind*’.

**ID 3087:** *‘I was creating paintings of things and situations I liked this helped me to appreciate things more and thus helped me transition to things. I seeked help from counsellor. I stayed in touch with my friends’.*

**ID 1515:** *‘Greater prioritising of activities that are better for physical and mental health over work. Keeping in touch with long-distance friends and family more often’.*

**ID 1779**: *‘I’m getting so bored working from home. I hate the isolation and feel so much less engaged. It’s so much harder for me to concentrate’.*

**ID 1155:** *‘It’s interfered with my efforts to break negatively reinforcing habits and has reinforced my sense of isolation and depression. I have gone from optimistic about the future to ambivalent at best’.*

**ID 792:** *‘zunehmende Antriebs- und Motivationslosigkeit, Desillusionierung über Kompetenzen und guten Willen der Regierung.’* [*increasing lack of drive and motivation, disillusionment with skills and goodwill of the government*.]

**ID 1314:** *‘overall pessimism on future-outlook, inability to plan ahead’.*

**ID 662;** *‘I have gotten significantly more negative and pessimistic especially when thinking about the future. Work–life balance has decreased significantly as we move to use online tools for work and school. concentration has definitely decreased too’.*

**ID 1164:** *‘Emotional fatigue from social isolation. Not able to travel to see parents in home country. Mental health definitely worsened. Became much more sedentary, gained weight, indulged in comfort eating. Became quite difficult to follow a healthy routine or schedule. Motivation was low from about 6 months into the pandemic and restrictions’.*

**ID 1708;** *‘Anxiety levels have increased, unable to motivate myself to do work, feeling lonely and not talking to friends when I feel sad, sleeping and eating have been very very irregular, feel a lot more hopeless and I don’t trust things to stick around anymore’.*

**ID 1188:** *‘Worried about my teaching contract not being extended’.*

**ID 3037:** *‘For everything to not be shut down again, or to at least figure out how to sell online and not need outdoor events to make money’.*

**ID 577:** *‘Able to work and handle childcare-being able to juggle a lot of things at once’.*

**ID 1138:** *‘Money: regular and unconditional so that I don’t waste brain time on job shit. I’m disabled and while I could work some jobs with support, I have never gotten all of what I need so I think that in the current situation, it makes sense for jobs to go to other people who could do them better. That doesn’t mean my partner and I could suddenly survive on air, however. I need therapy, so that also takes money; I need PIP for this, so I need help fighting the DWP for the disability support I’ve never had and still need. My partner needs therapy and job support too. My family needs disability support and childcare, especially while my stepdad is an essential worker. My friends desperately need money for living expenses, therapy and – my god – recreation to make life worth living’.*

**ID 24:** ‘*Many restrictions, not being able to do things/work/study (access to primary sources for my research)’.*

**ID 93:** ‘*Working from home has increased my workload and affected my motivation. Finally, homeschooling has been exhausting’.*

**ID 297:** *‘nature of work, workload and work pattern has completely changed my lifestyle is more isolated from others’.*

**ID 1408:** *‘My workload has increased a lot last year and I have job insecurities. It was hard to only focus on work and household duties without having opportunities for fun’.*

**ID 462:** *‘Increased workload, not enough time for hobbies/doing things for myself’.*

**ID 1729:** *‘My work (teaching) has been moved primarily online environment, which has resulted in my workload increasing by at least 50% in terms of effort and time and has degraded my ability to understand and respond to my student’s needs. I also am less hopeful about the future and find myself thinking about just surviving the next few years and not really planning anything new in my life’.*

**ID 827:** *‘A workload that isn’t crippling so I can spend more time with my son’.*

**ID 625:** *‘I learned how to deal with trauma memories thanks to my therapist. I attempted suicide in February and am now having sessions with a listening place around where I live’.*

**ID 1704:** *‘I started online therapy with a good therapist – knowing that this was going to be a rough ride. I think that helped me. I made it a project to learn how to cope better’.*

*People’s attitudes towards themselves and others have changed for the better and for worse*

**ID 1959:** *‘The pandemic has taught me just how little this government cares about the everyday person and important issues. It has become clear how unkind and insensitive most people are. Our health care system is broken. I have learnt that shared housing is terrible for mental health and people need pets. I have also learnt that there are other career options for me that I cannot access because of funding’.*

**ID 1511:** *‘Stress about Covid and how the government has handled it, including the handling of the vaccine rollout. Working from home I can now vape at my computer so I might vape slightly more’.*

**ID 41:** *‘I have asked for help so many times before and it’s just not available or the quality so poor as to make it ineffective. The public sector is now run by people who have no idea about working class life and the struggles people face. This leads to there being no help available when people really need it’.*

**ID 528:** *‘I will be for ever thankful for Marcus Rashford for the vouchers in lockdown made a huge difference and also a moral boost’.*

**ID 280:** *‘Financially, another stimulus check or two would be ideal. I kept my job but lost my supplemental income’.*

**ID 452:** *‘I spend less time with friends in the UK. I have not been able to travel to my home country and visit friends and family there as much as I would have before the pandemic. I do more yoga and exercise than before the pandemic. I go out less often, and I rarely go somewhere that requires travelling by public transport. I worry a bit more than I used to’.*

**ID 1811:** *‘being a single mom, struggled with childcare, started therapy, started therapy for my kids’.*

**ID 1223:** *’I need to buy a house real quick so I can move out. It has caused me so much mental stress I can’t even operate anymore. I have no support from anyone’.*

**ID 3050:** *‘Had to move house because of not getting along with my family member during lockdown’.*

**ID 1355:** *‘I have found that my family are not as supportive as they could be’.*

**ID 528:** *‘Prior to the pandemic we were already worn down by trying to live on £800 per month universal credit after years of being on zero hours and a redundancy. Sometimes the only way I could pay for shopping was to use PayPal as that took 3 days to clear. To not have food for your child is the most stressful thing’. ‘They [DWP] said they would refer us to a food bank but it was in the church at the end of my road and I felt so ashamed to go’.*

**ID 1138:** *‘can’t afford a therapist so my self-hatred has spiralled massively. I’ve been fighting with the DWP (Department for Work and Pensions) again to try for PIP (Personal Independence Payment) again, but every mental effort is so exhausting, and I have no idea where anything is, if I even have copies to begin with. I feel like other disabled people need more help, so when they ask for “proof” to support my “claim”, I don’t have any “evidence” from adult social care that I need help they’re probably too underfunded to give. Everything is too much and I’m angry that I have to tiptoe around saying how overwhelmed I am to avoid being sectioned, which would of course make everything better!’*

**ID 41:** *‘I learnt that I can trust those closest to me and with the help of an amazingly kind therapist got through my mother being ill and dying (I seriously don’t think I would have got through it otherwise.)’*

**ID 725:** *‘Trust in government has deteriorated, not sure if this government can properly run the country’.*

**ID 3301:** *‘I have been feeling so much anger towards my government and its administration. There are days when this anger would consume me’.*

**ID 2258:** *‘I feel much worse about the state of our country and about the selfish behaviours of other people. I hate reading the news because it always makes me sad’.*

**ID 847:** ‘*La mia fiducia nell’umanità è estremamente calata. Una pandemia poteva essere il nemico comune, quell’escamotage di cui l’umanità aveva bisogno per agire ed interagire come un sol popolo. Invece OGNI singolo ha pensato ad i propri interessi.* [*My confidence in humanity has dropped extremely’. A pandemic could be the common enemy, that ploy that humanity needed to act and interact as one people. Instead, EVERY individual has thought of their own interests*.]

**ID 1937:** *‘Only in regard to strengthening my belief that the majority of humans are self-obsessed and thoughtless. Think only of themselves and their wants in the very short term and give absolutely no thought to the environment or anything or anyone outside their immediate circle. There is no hope for the future of this planet when even a global pandemic can’t make those people think about more than themselves’.*

**ID 1923:** *‘Unfortunately I learnt that many of my acquaintances were idiots, posted Covid misinformation etc. on social media’.*

**ID 1216:** *‘the members of my household have been quite stressed during the pandemic and I have worked hard on taking time out for myself to unwind and not get caught up in the rollercoaster of other people’s emotions’.*

**ID 2057:** *‘I have learned that some people are selfish and that I don’t want to be connected to them, but I’ve learned that I have a fantastic support system that I appreciate very much’.*

**ID 3087: ‘** *I am a lot more irritable now that I am staying with my parents who bicker a lot but somehow are happy. The conflicts/previous issues that I believe sorting are causing significant amount of stress too. Like they have become unavoidable too’.*

**ID 1907:** *‘I am more appreciative of being able to spend time with friends and family’.*

**ID 1771:** *‘I have learnt how to take care of myself better - what makes me feel good when I’m off, what habits make my day better and how to deal with things a bit more on my own’.*

**ID 1188:** *‘I learned to appreciate the time together with my toddler and to emphasize my own mental health. This is something I carry into my teaching. I have completely revised my course policies to emphasize mental health and compassionate teaching as a result of my own and my students’ experiences during the pandemic. I think Covid-19 has made me a better teacher and a better human being in the classroom’.*

**ID 1675:** *‘Covid-19 pandemic brought significant changes in terms lifestyle, behaviour and thinking. Explicitly, it allowed me a full resetting – from am overambitious person, with lots of professional responsibilities and a quite stressful life, I am now a person focusing on family life, healthy lifestyle, inner peace, etc. The pandemic gave me the opportunity to clearly see what is really essential in my life and determined me to focus on that’.*

**ID 3403:** ‘各有利弊吧。也算是一种新的学习和生活方式，且在这个过程中确实找到了自己更喜欢的未来方向。除了学术，在生活和财务上反而是有所好转的（一直都不喜欢出门），在这个过程中也学到了一些事情’. [*Each has its pros and cons. It can be regarded as a new study and lifestyle, and in the process, I have indeed found a future direction I prefer. In addition to academics, my life and finances have improved (I have never liked to go out), and I have learned a few things in the process.*]

**ID 434:** *‘Opportunities to develop and grow, and patience to get there. Allowing more time to get back on track and find motivation. It takes a lot more to motivate me at the moment but I know it’s still in there’.*

*People’s mental and physical health have been primarily negatively impacted by the Covid-19 with some positive impacts*

**ID 1313:** *‘constant anxiety and less resilience’.*

**ID 1539:** ‘*More antisocial than ever before, panic attacks in shops etc., gained weight from not going to the gym etc., don’t want people to touch or even stand near me even if I know them, I’m sure it’ll take years to undo some of the behaviours I’ve now learnt over the course of the pandemic. Being around people without panicking seems impossible*’.

**ID 1355:** ‘*I have had deterioration in mental health including psychosis and self-harm. My eating disorder, anorexia has retriggered. I am craving cannabis. I feel afraid in public places, and I avoid going anywhere with crowds. I am quite socially isolated. My daughter has cut off all communication with me, so I feel hurt, angry and unsupported*’.

**ID 847:** ‘*Il mio peccato capitale è la pigrizia e il lockdown mi ha solo permesso di indulgere nell’accidia*’*.* [*My cardinal sin is laziness, and the lockdown has only allowed me to indulge in sloth.*]

**ID 235:** ‘*Self-funding and starting to attend trauma therapy, which has been life changing in so many ways and has definitely helped me to survive one of the most challenging times of my life as well as to finally have a professional relationship that is focused on validating me and my experiences, being understood and someone actually believing, and as such helping me to believe, that I can progress to recovery and what this means (not a cure but a chance to not let the past control my life like it has for so long)*’.

**ID 60:** ‘*Worse in the sense of anxiety and having to manage alone without having the physical support of family who do not live near me. Worse in that is has restricted physical activities and school, which has impacted on my oldest child who has autism and has struggled as she has lost her routines, been restricted in activities and contact with school and wider family. Worse in the sense of anxiety due to the incompetence of central government, lack of transparency and accountability, lack of media openness and the corruption of central government around PPE contracts etc, impact on the NHS waiting lists and services provided, impact on health and care workers, and sense of loss for people who I work with who have had to shield*’.

**ID 149:** ‘*For the first time I am starting to feel lonely. I miss the social interaction I got from being at work (my business has us all working from home still). My mental health has deteriorated slightly as a result*’.

**ID 439:** ‘*My mental health was worse during lockdown. This has improved as life is very much back to normal in my country*’.

**ID 485:** ‘*I’ve had a lot of anxiety about what the right thing is to do in different situations. Even though I think we are more cautious than many people, I worry about the risks we do take and their impact on the community. I have remained fairly active but I do sit more and walk much less now that I don’t commute. My mental health has fluctuated with some bouts of anxiety or depression*’.

**ID 780:** ‘*Mental health is very fragile. I need to consciously make an effort every day to feel ‘okay’. Little things will set me off crying*’*.*

**ID 807:** ‘*It was already hard to socialise (in-person) prior to the pandemic, but now there’s another layer of stress caused by my focus on social distancing. My mental health was fine prior to the pandemic, but now it’s certainly not. I used to be more physically active, but that has dropped. My decreasing physical health throughout the pandemic (partly caused by and partly *causing* the decreased physical activity) is not good*’.

**ID 1032:** ‘*Made me more able to put up with boring circumstances, mental health improved as I had a chance to practice coping mechanisms in a more sterlised environment, made me value my friendships and freedom more*’.

**ID 1188:** ‘*I learned to appreciate the time together with my toddler and to emphasize my own mental health. This is something I carry into my teaching. I have completely revised my course policies to emphasize mental health and compassionate teaching as a result of my own and my students’ experiences during the pandemic. I think Covid-19 has made me a better teacher and a better human being in the classroom*’.

**ID 1658:** ‘*Isolated. Increased social anxiety now there are lots of people out again. Gained weight. Boredom eating. Lazy. More worried if I don’t hear from some people for while. Paranoid. Really missed meeting up with friends in the week for lunch. Sleep more in the day. Prevented me from going to mental health support places in the day. Being judged eg if not wearing a mask*’.

**ID 1690:** ‘*I had made me stress about the future, my mental health has declined. I feel alone and that nobody understands me*’.

## Appendix 6

**ID 761:** ‘*My level of underlying, constant stress has increased so that it’s always there. Even when I’m relaxed or not worrying about my work, I still get stressed anytime I have to leave my apartment to go pick up food or groceries or end up around people who aren’t wearing masks or social distancing. I get worried anytime a close relative decides to do something that seems unsafe, like go on a trip or to a large gathering. I get frustrated anytime a friend flaunts protective measures to travel, go to bars, host a party, or other generally unsafe behavior. I have a constant level of worry for the elderly people in my life, even if I know they’re being mostly safe. When people I know decide to reward themselves for “being good” or decide it’s safe to do something just because it’s something they really want to do without concern for other people, I have an immediate gut reaction of rage and frustration for the fact that I’ve spent most of the last year+ inside and haven’t even seen any friends or family members or travelled at all. Anytime someone invites me to a party or large gathering, I worry about how to decline the invitation and worry that they will see me as crazy or stupid, or just assume I’m living in fear when I’m actually trying to keep others safe. I’m extremely paranoid now of what others must think of me as I’m usually the only person in my circles actually following guidelines to keep both myself and others safe, and don’t really trust anyone anymore who I had previously thought were caring of others or smart enough to be safe. It has made me feel isolated and like I don’t truly have any friends, because only 1-2 of my friends have actually tried to follow safety guidelines, and the rest have selfishly done whatever they wanted when it was something fun they wanted to do or an event they didn’t want to miss out on. I’ve started to feel like I hate mostly everyone, and that just makes me hate myself. I don’t want to be so mad at everyone all the time, and now I feel mostly just jaded and bitter and everyone else’s lack of caring, which in turn has made me feel like I’m the one who is uncaring. I hate that caring for other people’s health and safety has made me an angry person, mostly due to how others have treated me. I get treated like I’m the selfish one for saying no to events and parties, or visits with family, but I’m terrified of someone I know or someone I don’t know getting Covid due to my actions. I don’t want to be responsible for community spread or any other person having horrible effects from Covid or developing a long term disability. I don’t even mind all that much not ever going out for work, socialization, travel, or shopping. What bothers me most is not feeling solidarity with others in my life or with my community. It always feels like I’m the only one even trying or doing anything for the sake of others, and constantly seeing everything get worse makes me feel like my efforts haven’t mattered or even achieved anything’.*

**ID 235:** ‘*That people who choose to be employed in the caring professions are not always “caring”. I’ve realised from going through the pandemic, who are my true friends and exactly what that means. I’ve realised that I am stronger than I ever thought but that despite making a huge amount of progress I need to build my support network, move somewhere that I finally be happy and safe and settled long-term and really work on building a positive future by setting and gradually achieving goals that I’ve dreamed of and put off for too long. I think the time has really come for me to fight to be able to have the life that I’ve longed for and deserved for so long, as if I don’t, I don’t think that I can continue with living the life/existence that I have been for too long because I haven’t realised that I am capable of so much more even with all of the obstacles in my path. I’ve learned to appreciate and be grateful for things a lot more, even the little things that I took for granted for so long. I’ve realised the importance of making creativity a priority in my life and I’ve realised that my dad is unlikely to ever change and that I need space and low expectations where he is concerned to prevent any further heartbreak being caused by him. I’ve realised that I want to make a difference and that I want to be a positive influence in the lives of people I come into contact with, whilst continuing to be honest and authentic. I’ve realised how much my mum means to me even more now that we have become closer than ever during the pandemic, even despite the distance, and it means so much for our relationship to finally be all I ever dreamed for it to be and more. Most of all I’ve realised that it’s during the darkest times that you notice any light at all, even if it’s just a distant glimmer, and the importance of focusing on those’.*

**ID 235:** ‘*To get as far away from South Wales, my abusive neighbour and the appalling public services who have not only enabled him but really impacted negatively on my mental health. If it wasn’t for them I would be much further along on my recovery journey than I am. I am hoping to relocate to England where hopefully there will be better services, attitudes, wellness and care for both my physical and mental health, that I can be physically closer to my family which is a huge safety factor for me and I hope that in time, as our relationship improves that I will be able to be more of a support to them. I will also have many more options of pursuing hobbies, interests, courses/education and perhaps some form of flexible voluntary or freelance work.*

*Better access to physical and mental health support and if necessary treatment, would be of huge impact to me and my family. The pandemic has put a lot of tests and in-person specialist appointments on hold or at the end of huge waiting lists. An example of this, my mother in her 60s has been waiting for a couple of months already for an urgent liver scan which then adds to stress and worry for her and our family as well as meaning that she has to continue to struggle with difficult symptoms whilst holding down a full-time job. I have developed an eating disorder and I need to have assessment and treatment by specialist mental health professionals that I can trust, an assessment for ocd and more support for my ptsd.*

*I think opportunities for people to access support groups both within and outside of the mental health team, as a lot of friendships/relationships have suffered.*

*Reassurance that the government can be relied upon to manage any concerning peaks in the Covid rates as early as possible with less intrusive methods rather than leaving things to continue and the UK needing to go into yet another long term pandemic, as I know that this would be a hugely negative experience for me and my entire family. I also think that as soon as possible there needs to be an independent enquiry, not just into how the pandemic could have been handled better (especially initially), why propaganda from anti-vaxxers, that has led to some people becoming fearful of the vaccine, has been allowed to spread so easily with very little of it being challenged quickly enough by a variety of trusted people (not politicians) and a clear communication of the facts. How many people have been affected by long-covid and what sorts of treatment/care/support they need and how this can be best provided.*

*And a really huge one for me (which in turn impacts my family hugely) is a report into how those with mental illness have been during the pandemic (studies like this will be incredibly useful for this), those who have developed or relapsed mental illnesses, how they have been treated (was treatment even available/accessible – especially for those who were shielding etc?), how many people have lost their lives during lock down as a result of mental illness, how many of these deaths could have been prevented? What could have helped? What needs to be put in place urgently and in the long-term to ensure that those suffering do not get left on waiting lists etc and things escalate unnecessarily? These are such important questions but I doubt the government and public services would be willing to be held accountable. My only hope is that if they do not undertake these enquiries themselves that, a probably better and, more likely trustworthy method, would be for a charity/charities or organisation/organisations will investigate this as I think it is so important for us to not just get caught up in the joy of returning to some form of normality and see this whole experience with Rose tinted glasses, while it is good to appreciate what was successful, it is always good to acknowledge that nothing is ever handled perfectly and so it is good to learn whether things can be done better/differently next time/in any similar situation for a more positive outcome*’.

## References

[1] World Health Organization (2020). WHO Director-General’s opening remarks at the 487 media briefing on COVID-19, 11 March 2020.

[2] Carollo A, Bizzego A, Gabrieli G, Wong K, Raine A, Esposito G (2021a). I’m alone but not lonely. U-shaped pattern of self-perceived loneliness during the COVID-19 pandemic in the UK and Greece. Public Health Pract.

[3] Carollo A, Bizzego A, Gabrieli G, Wong K, Raine A, Esposito G (2021b). Self-perceived loneliness and depression during the COVID-19 pandemic: a two-wave replication study. UCL Open Environ Preprint.

[4] Panchal U, Salazar de Pablo G, Franco M, Moreno C, Parellada M, Arango C (2021). The impact of COVID-19 lockdown on child and adolescent mental health: systematic review. Eur Child Adolesc Psychiatry.

[5] Singh S, Roy MD, Sinha CPTMK, Parveen CPTMS, Sharma CPTG, Joshi CPTG (2020). Impact of COVID-19 and lockdown on mental health of children and adolescents: a narrative review with recommendations. Psychiatry Res.

[6] Wang Y, Shi L, Que J, Lu Q, Liu L, Lu Z (2021). The impact of quarantine on mental health status among general population in China during the COVID-19 pandemic. Mol Psychiatry.

[7] McKibbin W, Fernando R (2021). The global macroeconomic impacts of COVID-19: seven scenarios. Asian Econ Pap.

[8] Rossi R, Socci V, Talevi D, Mensi S, Niolu C, Pacitti F (2020). COVID-19 pandemic and lockdown measures impact on mental health among the general population in Italy. Front Psychiatry.

[9] Saunders R, Buckman JE, Fonagy P, Fancourt D (2021). Understanding different trajectories of mental health across the general population during the COVID-19 pandemic. Psychol Med.

[10] Wang C, Pan R, Wan X, Tan Y, Xu L, McIntyre RS (2020). A longitudinal study on the mental health of general population during the COVID-19 epidemic in China. Brain Behav Immun.

[11] Braquehais MD, Vargas-Cáceres S, Gómez-Durán E, Nieva G, Valero S, Casas M (2020). The impact of the COVID-19 pandemic on the mental health of healthcare professionals. QJM.

[12] Gupta S, Sahoo S (2020). Pandemic and mental health of the front-line healthcare workers: a review and implications in the Indian context amidst COVID-19. Gen Psychiatr.

[13] Ozamiz-Etxebarria N, Idoiaga Mondragon N, Bueno-Notivol J, Pérez-Moreno M, Santabárbara J (2021). Prevalence of anxiety, depression, and stress among teachers during the COVID-19 pandemic: a rapid systematic review with meta-analysis. Brain Sci.

[14] Fond G, Nemani K, Etchecopar-Etchart D, Loundou A, Goff DC, Lee SW (2021). Association between mental health disorders and mortality among patients with COVID-19 in 7 countries: a systematic review and meta-analysis. JAMA Psychiatry.

[15] Portnoy J, Bedoya A, Wong K (2022). Child externalising and internalising behaviour and parental wellbeing during the Covid-19 pandemic. UCL Open: Environment.

[16] Ravens-Sieberer U, Kaman A, Erhart M, Otto C, Devine J, Löffler C (2021). Quality of life and mental health in children and adolescents during the first year of the COVID-19 pandemic: results of a two-wave nationwide population-based study. Eur Child Adolesc Psychiatry.

[17] Waite P, Pearcey S, Shum A, Jasmine R, Patalay P, Creswell C (2021). How did the mental health symptoms of children and adolescents change over early lockdown during the COVID-19 pandemic in the UK?. J Child Psychol Psychiatr Adv.

[18] Son C, Hegde S, Smith A, Wang X, Sasangohar F (2020). Effects of COVID-19 on college students’ mental health in the United States: interview survey study. J Med Internet Res.

[19] Sideropoulos V, Midouhas E, Kokosi T, Brinkert J, Wong K, Kambouri M (2021). The effects of cumulative stressful educational events on the mental health of doctoral students during the COVID-19 pandemic. UCL Open Environment Preprint.

[20] Wong K, Wang Y, Esposito G, Raine A (2021). A three-wave network analysis of COVID-19’s impact on schizotypal traits, paranoia and mental health through loneliness. UCL Open Environ Preprint.

[21] Varga TV, Bu F, Dissing AS, Elsenburg LK, Bustamante JJH, Matta J (2021). Loneliness, worries, anxiety, and precautionary behaviours in response to the COVID-19 pandemic: a longitudinal analysis of 200,000 Western and Northern Europeans. Lancet Reg Health-Eur.

[22] McKinlay AR, Fancourt D, Burton A (2021). A qualitative study about the mental health and wellbeing of older adults in the UK during the COVID-19 pandemic. BMC Geriatr.

[23] Pisula P, Salas Apaza JA, Baez GN, Loza CA, Valverdi R, Discacciati V (2021). A qualitative study on the elderly and mental health during the COVID-19 lockdown in Buenos Aires, Argentina – Part 1. Medwave.

[24] O’Sullivan K, Clark S, McGrane A, Rock N, Burke L, Boyle N (2021). A qualitative study of child and adolescent mental health during the COVID-19 pandemic in Ireland. Int J Environ Res Public Health.

[25] Sideropoulos V, Dukes D, Hanley M, Palikara O, Rhodes S, Riby DM (2022). The impact of COVID-19 on anxiety and worries for families of individuals with special education needs and disabilities in the UK. J Autism Dev Disord.

[26] Gillard S, Dare C, Hardy J, Nyikavaranda P, Olive RR, Shah P (2021). Experiences of living with mental health problems during the COVID-19 pandemic in the UK: a coproduced, participatory qualitative interview study. Soc Psychiatry Psychiatr Epidemiol.

[27] Ardebili ME, Naserbakht M, Bernstein C, Alazmani-Noodeh F, Hakimi H, Ranjbar H (2021). Healthcare providers experience of working during the COVID-19 pandemic: a qualitative study. Am J Infect Control.

[28] Mortensen CB, Zachodnik J, Caspersen SF, Geisler A (2022). Healthcare professionals’ experiences during the initial stage of the COVID-19 pandemic in the intensive care unit: a qualitative study. Intensive Crit Care Nurs.

[29] De Leo A, Cianci E, Mastore P, Gozzoli C (2021). Protective and risk factors of Italian healthcare professionals during the COVID-19 pandemic outbreak: a qualitative study. Int J Environ Res Public Health.

[30] Braun V, Clarke V (2006). Using thematic analysis in psychology. Qual Res Psychol.

[31] Han Q, Zheng B, Cristea M, Agostini M, Bélanger JJ, Gützkow B (2021). Trust in government regarding COVID-19 and its associations with preventive health behaviour and prosocial behaviour during the pandemic: a cross-sectional and longitudinal study. Psychol Med.

[32] Ali F, Russell C, Nafeh F, Rehm J, LeBlanc S, Elton-Marshall T (2021). Changes in substance supply and use characteristics among people who use drugs (PWUD) during the COVID-19 global pandemic: a national qualitative assessment in Canada. Int J Drug Policy.

[33] Andriyani FD, Biddle SJ, De Cocker K (2021). Adolescents’ physical activity and sedentary behaviour in Indonesia during the COVID-19 pandemic: a qualitative study of mothers’ perspectives. BMC Public Health.

[34] Petersen JA, Naish C, Ghoneim D, Cabaj JL, Doyle-Baker PK, McCormack GR (2021). Impact of the COVID-19 pandemic on physical activity and sedentary behaviour: a qualitative study in a Canadian city. Int J Environ Res Public Health.

[35] Chen H, Shi L, Zhang Y, Wang X, Jiao J, Yang M (2021). Response to the COVID-19 pandemic: comparison of strategies in six countries. Front Public Health.

[36] Alanezi F, Aljahdali A, Alyousef SM, Alrashed H, Mushcab H, AlThani B (2020). A comparative study on the strategies adopted by the United Kingdom, India, China, Italy, and Saudi Arabia to contain the spread of the COVID-19 pandemic. J Healthc Leadership.

